# Neonatal sepsis in a tertiary unit in South Africa

**DOI:** 10.1186/s12879-021-05869-3

**Published:** 2021-02-27

**Authors:** Dharshni Pillay, Lerusha Naidoo, Khine Swe Swe-Han, Yesholata Mahabeer

**Affiliations:** 1 Department of Medical Microbiology, National Health Laboratory Service, Inkosi Albert Luthuli Central Hospital, 800 Vusi Mzimela Road, Durban, KwaZulu-Natal 4091 South Africa; 2 School of Laboratory Medicine and Medical Sciences, Nelson R. Mandela School of Medicine, University of KwaZulu Natal, 716 Umbilo Road, Berea, KwaZulu-Natal 4001 South Africa; 3 Neonatal Intensive Care Unit, Inkosi Albert Luthuli Central Hospital, 800 Vusi Mzimela Road, Durban, KwaZulu-Natal 4091 South Africa; 4 Department of Paediatrics and Child Health, Nelson R Mandela School of Medicine, University of KwaZulu Natal, 716 Umbilo Road, Berea, KwaZulu-Natal 4001 South Africa

**Keywords:** Neonatal sepsis, Microbial profiles, Antimicrobial resistance, Empiric regimens

## Abstract

**Background:**

Antimicrobial resistance (AMR) has emerged as a global threat to healthcare resulting in an increase in morbidity and mortality. Neonatal sepsis is ranked as the third highest cause of neonatal demise globally, in which AMR accounted for 31.0% of deaths. AMR in neonates has been poorly characterised in Durban, South Africa. Thus, the resultant effect of AMR on empiric regimens for neonatal sepsis is uncertain in this setting. Therefore, this study analysed the aetiology and antimicrobial susceptibility patterns of bloodstream infections within the neonatal intensive care unit at a tertiary hospital in Durban, with the aim of establishing an effective empiric regimen for the unit.

**Methods:**

A retrospective data review on positive blood cultures from the neonatal intensive care unit at Inkosi Albert Luthuli Central Hospital was conducted. Three time periods were analysed: 2014, 2016 and 2018. Culture data from neonates aged 0–30 days were included and repeat cultures were de-duplicated. The frequency of common organisms and their antimicrobial susceptibilities were analysed. Fischer’s exact test was used for subgroup analysis. Poisson and logistic regressions were used to assess significant trends in organisms and antimicrobial susceptibilities over time.

**Results:**

Late-onset sepsis (86.8%) predominated over early-onset sepsis (13.2%). A preponderance of gram-positive organisms (68.7%) over gram-negatives (26.8%) and fungi (4.5%) was detected. Common pathogens included coagulase-negative staphylococci (53.5%), *Klebsiella pneumoniae* (11.6%)*,* enterococci (9.3%), and *Acinetobacter baumannii* (7.7%). Despite the small contribution of fungi to the microbial profile, fluconazole-resistant *Candida parapsilosis* predominated within that group. High rates of resistance to first- and second-line antibiotics were also noted among gram-positive and gram-negative organisms. Multidrug resistant organisms included extended-spectrum beta-lactamase (ESBL) *K. pneumoniae* (7.6%) and extensively-drug resistant *A. baumannii* (7.0%). However, a statistically significant decrease in ESBL-producing organisms was documented during the entire study period (*p* = 0.005).

**Conclusions:**

It was determined that first-line antimicrobials, advocated by the World Health Organization for treatment of neonatal sepsis, proved ineffective in this unit due to high levels of AMR. Therefore, this study advises that meropenem with or without vancomycin provides optimal empiric cover. Amphotericin B is advocated for empiric antifungal therapy. Ongoing surveillance is necessary.

## Introduction

Antimicrobial resistance (AMR) has emerged as a global threat to healthcare resulting in an increase in morbidity and mortality [1]. An estimated 31.0% of deaths from neonatal sepsis are attributed to AMR [2]. Sepsis accounts for 6.8% of neonatal deaths, ranking it as the third highest cause of neonatal demise following preterm births and intrapartum-related events [3]. In Sub-Saharan Africa, sepsis-related neonatal mortality rates are high and range between 17.0 to 29.0% [4].

Neonatal sepsis presents unique diagnostic challenges largely due to the absence of a universal definition [5]. Traditionally defined as sepsis within the first 28 days of life, neonatal sepsis can be further stratified into early-onset (< 3 days) and late-onset (≥ 3 days) [6]. It entails a collection of non-specific clinical features or laboratory signs of sepsis with positive microbiological cultures from a sterile sample (although cultures may sometimes be negative). A significant isolate from a blood culture is the gold standard for diagnosis [6].

Based on blood culture data, early-onset sepsis (EOS) and late-onset sepsis (LOS) differ in microbial profiles. Group B streptococcus (GBS) is a major cause of early onset sepsis in high-income countries (HIC) [7]. This contrasts with the bacteriological profile of resource-limited settings where GBS rates are lower and *Klebsiella pneumoniae*, *Staphylococcus aureus* and coagulase-negative staphylococci (CoNS) are the predominant pathogens of EOS [8, 9]. In South Africa, EOS is mainly caused by GBS*, K. pneumoniae* and *Escherichia coli* [10–12].

A wider spectrum of gram-negatives, including *K. pneumoniae*, *Citrobacter* species, *E. coli, Enterobacter* species, *Acinetobacter baumannii* and *Pseudomonas aeruginosa* are observed in LOS [13, 14]*.* Furthermore, CoNS cause significant cases of gram-positive LOS in many countries [11, 15]. Other important bacterial causes of LOS in South Africa are enterococci, *K. pneumoniae* and *Acinetobacter* species [12, 16].

A strong association between fungal infections and LOS suggests a causal relationship with hospital intervention [17]. Globally, *Candida* species have been reported as the third leading cause of blood-stream infection (BSI) amongst extremely-low-birthweight neonates and in South Africa, *C. parapsilosis* is a leading cause of candidaemia in neonates [18].

Global resistance to first-line empiric treatment regimens is on the increase [8, 13, 19]. This NICU recommends empiric antibiotic regimens according to timing of sepsis. Amoxicillin-clavulanate is used for EOS and piperacillin-tazobactam plus amikacin for LOS. Escalation to meropenem depends on the therapeutic response with the addition of vancomycin and fluconazole in the presence of risk factors.

Multi-drug resistance adds a further complication to antimicrobial choices. South African studies have documented the emergence of drug resistance to multiple antibiotics, including extended-spectrum β-lactamase production (ESBL) and methicillin-resistant *S. aureus* (MRSA) in neonates [10, 12, 16, 20]. These changing patterns of resistance require regular microbial surveys be conducted. Consequently, understanding the microbial profile of a neonatal unit contributes significantly to early appropriate empiric antimicrobial choices in the management of sepsis. This improves therapeutic outcomes and reduces mortality. Notably in KwaZulu-Natal (which encompasses Durban), a limited scientific data base surrounding local microbial profiles and AMR in neonatal sepsis exists. Therefore, this study aims to establish the microbiological and antimicrobial susceptibility profiles and trends of neonatal bloodstream infections in the NICU of Inkosi Albert Luthuli Central Hospital to guide empiric antimicrobial management of EOS and LOS.

## Methods

### Study design, location, and period

This study is a retrospective review of positive blood cultures from the NICU at IALCH, which is a tertiary and the only quaternary referral unit for the province of KwaZulu-Natal, South Africa. The unit consists of 12 intensive care and 8 high-care beds, with approximately 700 to 800 admissions per year and a bed occupancy rate of 100%. The patients are usually from surgical, neurosurgical and cardiology disciplines.

Data was collected from 2014 to 2018 at three biennial periods: 2014, 2016 and 2018. The data was accessed from the National Health Laboratory Service (NHLS) Central Data Warehouse.

### Study population

The study samples consisted of all positive blood cultures from the NICU for the period January to December in years 2014, 2016 and 2018. Individual episodes of sepsis were sought for the study population. Samples were included from patients aged 0 to 30 days of life. Repeat blood cultures taken within 14 days of the index culture, where the same organism was isolated again, were excluded from the study.

### Laboratory methods and information handling

The general techniques utilised by the laboratory to generate the microbial identifications and antimicrobial susceptibility testing include manual methods such as Kirby-Bauer disk diffusion. Automated identifications and susceptibility testing were performed using Vitek 2 Advanced Expert System™ (bioMerieux). Data was entered onto the laboratory information system which interfaces with the Central Data Warehouse.

### Measurements

The onset of sepsis was classified as early-onset (< 3 days old) and late-onset sepsis (≥ 3 days old) measured from the date of birth until collection of the index culture.

Primary outcomes:

1. The frequency of common organisms and antimicrobial susceptibility patterns were evaluated as a proportion of the total number of positive cultures. Antimicrobial susceptibility patterns were stratified and analysed according to gram-positive, gram-negative, and fungal antimicrobial panels. These panels consisted of antimicrobials routinely tested within the IALCH microbiology laboratory.
2. Trends in prevalence of organisms and antimicrobial susceptibilities per year were calculated and prevalence of specific multi-drug resistant (MDR) organisms during the study period was determined. These MDR organisms included [21]:

- MDR gram-negatives – resistant to at least one agent from two or more classes of all tested antimicrobial agents.
- XDR - non-susceptibility to one or more agents in two or less categories of antimicrobials.
- Carbapenem-resistant Enterobacterales (CRE) – resistant to at least one carbapenem (imipenem, meropenem, ertapenem).
- ESBL – resistant to a third or fourth generation cephalosporins or detected as an ESBL through automated methods (Vitek 2 Advanced Expert System™, bioMerieux)
- MRSA – *S. aureus* resistant to cloxacillin.
- VRE – enterococci resistant to vancomycin.

### Statistical analysis

Descriptive statistics were used to summarise the data. Categorical data were summarised by frequencies and percentage. The frequency of selected organisms was reported by year. Susceptibility of each drug was reported as the percentage susceptible. Frequency was calculated as a measure of the total number of samples in the data series. The number of each organism seen per year is a count variable. Comparisons of pathogens by subgroup, such as early-onset and late-onset neonatal sepsis, was done using Chi Square or Fisher’s exact test. Temporal trends in the number of organisms and antimicrobial susceptibility patterns over time were analysed using Poisson and logistic regression. Stata V13.1 was used for the data analysis and *p*-value of 0.05 was considered statistically significant.

### Ethical considerations

This study has received approval from the University of KwaZulu-Natal Biomedical Research Ethics Committee (BE019/19), IALCH, NHLS and the KwaZulu-Natal Department of Health.

## Results

**Fig. 1:**
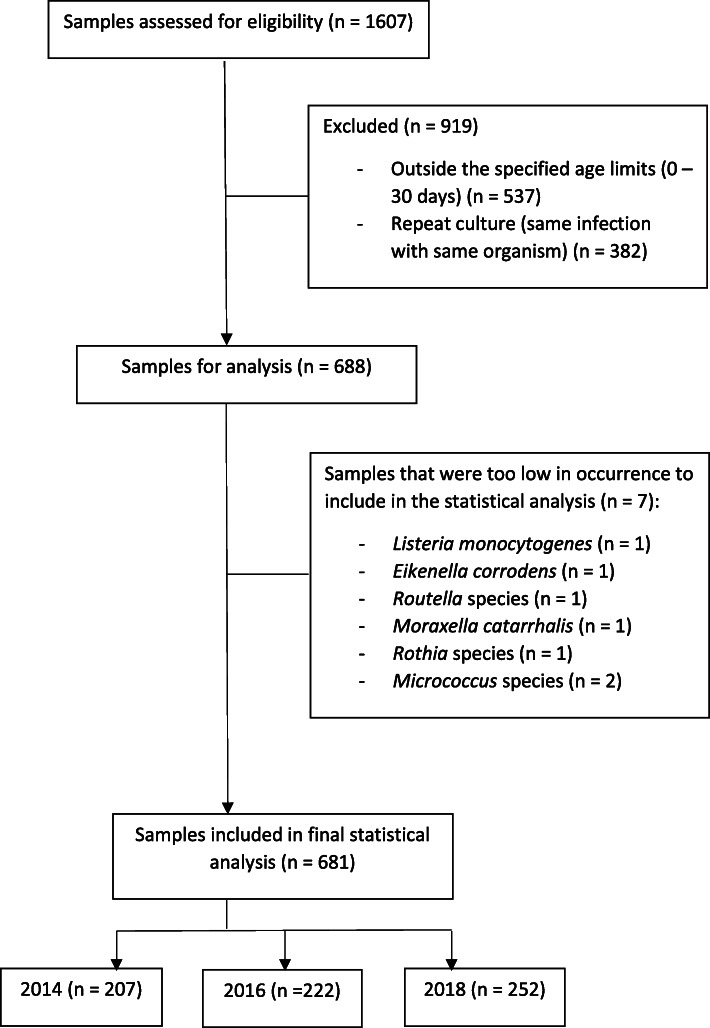
Derivation of study sample

As demonstrated in Figs. 1, 1607 isolates were initially assessed for eligibility during the three study periods. The final analysis included 681 isolates from 3 years: 2014 (207 isolates), 2016 (222 isolates) and 2018 (252 isolates). These were stratified into 3 groups: gram-positives (*S. aureus,* CoNS*, Streptococcus* species, *Enterococcus* species), gram- negatives (Enterobacterales, non-fermenting gram-negative organisms) and fungi.

### Overall microbial profile

Gram-positive organisms were predominantly isolated in this study (468/681; 68.7%). Within the gram-positive category, the main organisms were CoNS (363/468; 77.6%), *Enterococcus* species (64/468; 13.7%), *S. aureus* (24/468; 5.1%) and *Streptococcus* species (17/468; 3.6%). Often CoNS are common blood culture contaminants and in the absence of clinical correlation the significance of these organisms is unclear. If CoNS were excluded from the analysis enterococci emerge as the leading cause of gram-positive sepsis (64/105; 61.0%), comprising *Enterococcus faecium* (40/64, 62.5%); *Enterococcus faecalis* (22/64; 34.4%) and *Enterococcus* species (2/64; 3.1%). Only one *Streptococcus agalactiae* isolate was found during the study period.

Gram-negatives accounted for 26.8% of the total study population (182/681) which were divided into Enterobacterales (118/182; 64.8%) and non-fermenters (64/182; 35.1%). Within the Enterobacterales family, the most common organisms were *K. pneumoniae* (79/118; 66.9%), *E. coli* (13/118; 11.0%) and *Serratia marcescens* (10/118; 8.5%). *Acinetobacter baumannii* (53/64; 82.8%) accounted for greater than 80% of non-fermenters followed by *Stenotrophomonas maltophilia* (6/64; 9.4%) and *Pseudomonas aeruginosa* (5/64; 7.8%).

Fungal isolates were less commonly isolated than bacterial isolates (4.5% vs. 95.5%) and consisted of *C. parapsilosis* (14/31; 45.2%), *C. albicans* (9/31; 29.0%) and other *Candida* species that were not speciated further (8/31; 25.8%). Non-albicans *Candida* species (22/31; 71.0%) predominated over *C. albicans* (9/31; 29.0%).

### Microbial profile trends over 2014, 2016 and 2018

As summarised in Table 1, coagulase-negative staphylococci (53.2%), *K. pneumoniae* (11.5%), *A. baumannii* (7.8%) and *E. faecium* (5.9%) were the leading organisms across the study period and predominated within each analysed year. An increase in the number of enterococci (*E. faecium and E. faecalis)* was observed over time, and although not statistically significant, by 2018, it was the commonest organism in the unit after CoNS. Analysis of the trends of the other organisms revealed no significant patterns, except for *Streptococcus* species. *Streptococcus* species, specifically viridans streptococci, demonstrated a significant increase in the number of isolates between 2014 and 2018 (IRR 9. 04; 95% CI [1.17, 69.99]; *p* = 0.04). However, their overall contribution to the sample pool was low, 16/681 (1/16 in 2014, 4/16 in 2016 and 11/16 in 2018), therefore, there was no impact on the leading pathogens over the three study years.

**Table 1:** Trend in leading pathogens over the three study periods (2014, 2016, 2018)

Year	Rank	Organisms	Percentage
**2014**	1	CoNS	53.0
	2	*K. pneumoniae*	14.4
	3	*A. baumannii*	9.0
	4	*Enterococcus* species	9.0
**2016**	1	CoNS	51.4
	2	*K. pneumoniae*	16.7
	3	*Enterococcus* species	7.6
	4	*A. baumannii*	6.7
**2018**	1	CoNS	55.2
	2	*Enterococcus* species	11.5
	3	*K. pneumoniae*	9.9
	4	*A. baumannii*	5.1

### Early-onset sepsis versus late-onset sepsis

In this study, the majority of organisms were isolated during late-onset sepsis (591/681, 86.8%), with early-onset sepsis (EOS) accounting for only 13.2% (90/681) of cases (*p* = 0.02). As stipulated in Table 2, *S. aureus* and *E. faecalis* were more significant in EOS (*p* = 0.006 and *p* = 0.048, respectively). *E. faecium* emerged as an important gram-positive organism of LOS (*p* = 0.2). In addition, although *A. baumannii* is a predominant gram-negative organism in EOS, more *K. pneumoniae* isolates emerged in LOS. However, this difference was not of statistical significance.

**Table 2:** Leading pathogens in early-onset sepsis versus late-onset sepsis

	Early-onset sepsis (< 3 days)		Late-onset sepsis (≥ 3 days)	
Rank	Organism	Percent	Organism	Percent
**1**	CoNS	56.7	CoNS	52.8
**2**	*A. baumannii*	7.8	*K. pneumoniae*	12.4
**3**	*K. pneumoniae*	6.7	*A. baumannii*	7.8
**4**	*E. faecalis*	6.7	*E. faecium*	6.6
**5**	*Staphylococcus aureus*	6.7		

### Overall antimicrobial susceptibility

Among the gram-positive organisms, the susceptibilities to first-line therapies (i.e., ampicillin and cloxacillin) were low. However, all gram-positive isolates tested during the study period were susceptible to vancomycin (100.0%).

Gram-negatives also demonstrated high levels of resistance to commonly used first- and second-line antibiotics. Enterobacterales susceptibility to cefotaxime (34.2%) and piperacillin-tazobactam (55.5%) was low. However, susceptibility to carbapenems was maintained at greater than 90.0%. Additionally, this order demonstrated a higher susceptibility to amikacin than gentamicin (70.3% vs 39.7%, respectively).

Analysis of the non-fermenters, demonstrated low levels of susceptibility towards all tested antibiotics, including third-line agents: meropenem (17.2%), imipenem (13.5%) and ciprofloxacin (19.7%). The highest susceptibility was noted for amikacin (49.1%).

Approximately half the fungal isolates tested were susceptible to fluconazole, the first-line antifungal agent used in the unit.

Table 3 demonstrates the antimicrobial susceptibilities analysed over the study period.

**Table 3:** Overall antimicrobial susceptibility and trends for 2014, 2016 and 2018

Antimicrobials	2014 n (%)	2016 n (%)	2018 n (%)	Total study period - n (%)	Episodes omitted^**a**^ (n)
**Gram positive organisms**					
***Staphylococcus aureus***					
Cloxacillin	0/4 (0.0)	4/10 (40.0)	1/10 (10.0)	5/24 (20.8)	0
Vancomycin	3/3 (100.0)	10/10 (100.0)	9/9 (100.0)	22/22 (100.0)	2
**CoNS**					
Cloxacillin	5/100 (4.5)	13/114 (10.4)	14/139 (11.1)	32/363 (8.8)	0
Vancomycin	11/11 (100.0)	14/14 (100.0)	21/21 (100.0)	46/46 (100.0)	317
***Enterococcus*** **species**					
Ampicillin	6/18 (33.3)	7/17 (41.2)	11/29 (37.9)	24/64 (37.5)	0
Vancomycin	18/18 (100.0)	17/17 (100.0)	29/29 (100.0)	64/64 (100)	0
**Gram negatives organisms**					
**Enterobacterales**					
Amikacin	37/41 (90.2)	30/43 (38.6)	27/33 (57.6)	94/117 (80.3)	1
Cefotaxime	10/41 (24.4)	13/43 (30.2)	17/33 (51.5)	40/117 (34.2)	1
Ceftazidime	10/40 (25.0)	13/43 (30.2)	18/33 (54.5)	41/116 (35.3)	2
Ciprofloxacin	22/41 (53.7)	31/44 (70.5)	20/32 (62.5)	73/117 (62.4)	1
Ertapenem	33/34 (97.1)	34/38 (89.5)	26/31 (83.9)	93/103 (90.3)	15
Gentamicin	10/39 (25.6)	17/44 (38.6)	19/33 (57.6)	46/116 (39.7)	2
Imipenem	34/34 (100.0)	39/43 (90.7)	28/33 (84.8)	101/110 (91.8)	8
Meropenem	40/41 (97.6)	39/43 (90.7)	28/33 (84.8)	108/11792.3	1
Piperacillin-Tazobactam	23/41 (56.1)	21/39 (53.8)	17/30 (56.7)	61/11055.5	8
**Gram-negative non-fermenters**					
Amikacin	16/21 (76.2)	9/17 (52.9)	1/15 (6.7)	26/53 (49.1)	11
Ceftazidime	1/21 (4.8)	7/21 (33.3)	1/16 (6.3)	9/59 (15.3)	6
Ciprofloxacin	5/22 (22.7)	6/22 (27.3)	1/17 (5.9)	12/61 (19.7)	3
Gentamicin	2/22 (9.1)	3/17 (17.6)	1/13 (7.7)	6/52 (11.5)	12
Imipenem	2/16 (12.5)	5/20 (25)	0/16 (0.0)	7/52 (13.5)	12
Meropenem	5/22 (27.2)	5/20 (25.0)	0/16 (0.0)	10/58 (17.2)	6
Piperacillin-Tazobactam	4/22 (18.2)	4/20 (20.0)	1/16 (6.3)	6/58 (15.5)	6
**Fungal isolates**					
Fluconazole	2/9 (22.5)	7/10 (70.0)	18/12 (54.8)	17/31 (54.8)	0

^**a**^Omitted due to missing data

### Antimicrobial susceptibility trends over 2014, 2016 and 2018

Susceptibility during 2014, 2016 and 2018, for ciprofloxacin, ceftazidime, imipenem, meropenem, penicillin, ampicillin, piperacillin/tazobactam, vancomycin was static. However, a combined analysis of all the gram-negatives demonstrated a significant decrease in susceptibility of amikacin between 2014 (85.8%), 2016 (65.0%) and 2018 (53.8%) (OR 0.24; 95% CI [0.10, 0.59]; *p* = 0.002). During the same period, there was an increase in gentamicin susceptibility (19.7 to 43.4%; OR 3.14; 95% CI [1.33, 7.42]; *p* = 0.01). Another notable finding was a statically significant increase in cefotaxime susceptibility for Enterobacterales between 2014 (24.4%), 2016 (21.7%) and 2018 (55.1%) (OR 3.29; 95% CI [1.15, 7.09]; *p* = 0.02).

### Multidrug resistant organisms

**Fig. 2:**
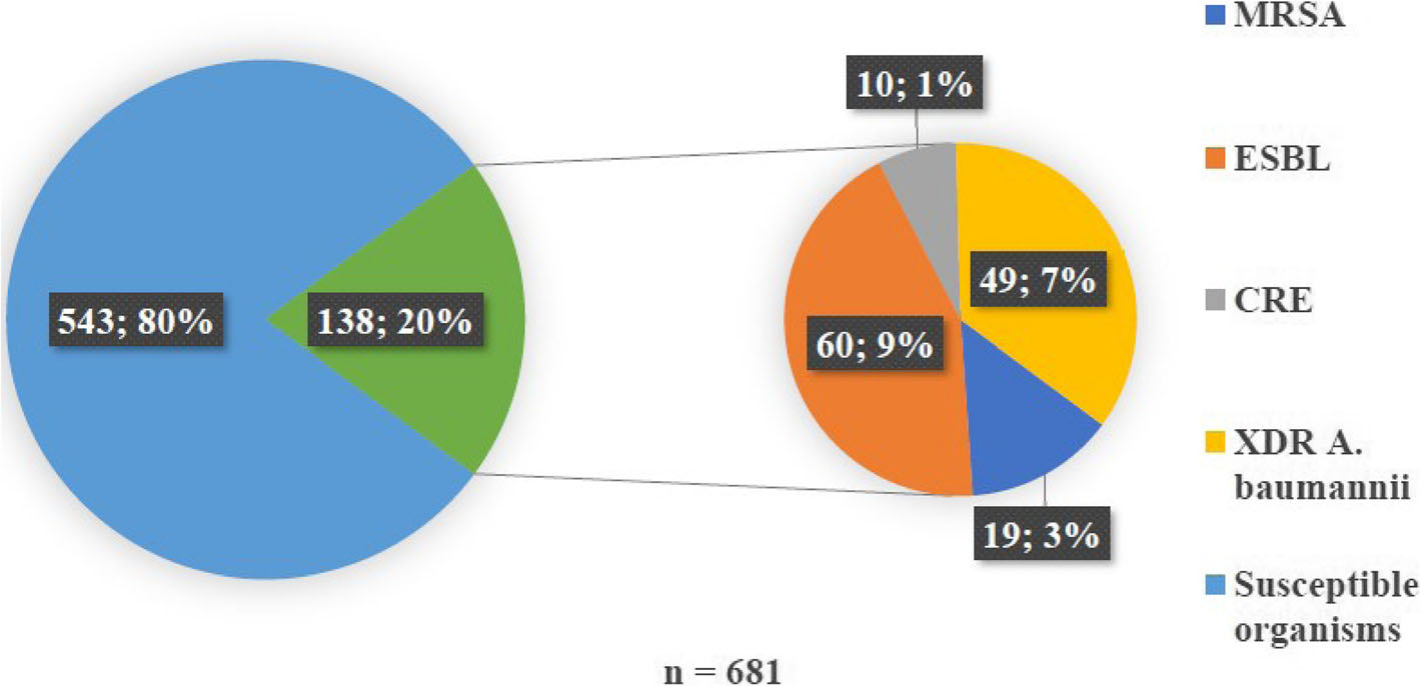
Multidrug resistant organisms during the study period

Figure 2 describes the composition of the MDROs, which constituted 20.0% of the total sample population (138/681).

ESBL organisms formed the majority of the MDROs in the unit (60/681; 9%) and were comprised of *K. pneumoniae* (52/681; 8%), with a minority of ESBL *E. coli* (8/681; 1%). Extensively-drug resistant (XDR) *A. baumannii* constituted 7.0% of the total resistance observed in the study. Overall, CREs accounted for 1.4% (10/681) of MDROs.

Most *S. aureus* isolates were MRSA (19/24; 79.0%) which comprised 3.0% (24/681) of the total study population.

When MDR organisms were analysed, a statistically significant decrease in all ESBL organisms was noted between 2014 (70.0%) and 2018 (36.4%) (OR 0.24; 95% CI [0.09, 0.65]; *p* = 0.005).

An increase in XDR isolates between 2014 (75.0%) and 2018 (88.9%) was detected, however, this was not of statistical significance (OR 2.67; 95% CI [0.47, 15.14]; *p* = 0.3).

Comparisons of CRE samples from 2014 (2.5%) to 2018 (15.2%) (OR 6.96; 95% CI [0.77, 62.93]; *p =* 0.08) demonstrated a non-significant increase in the number of cases.

## Discussion

This study represents the first published microbial profile of neonatal sepsis at a tertiary/quaternary unit in KwaZulu-Natal, South Africa. A preponderance of LOS versus EOS was observed. The leading organisms were CoNS, followed by *K. pneumoniae*, *A. baumannii* and *E. faecium.* A decrease in susceptibility to first- and second-line antibiotics, in gram-positives, gram-negatives and fungi was demonstrated. However, susceptibility to broad spectrum antibiotics such as vancomycin for gram positives and carbapenems for Enterobacterales was maintained. ESBL *K. pneumoniae* and XDR *Acinetobacter baumannii* represented the predominant MDR types.

Tertiary NICU settings, such as the NICU at IALCH, treat neonates that are mostly premature and of a low birth weight [22]. According to Giannoni et al. (2018), hospital-acquired LOS was higher in preterm infants when compared to EOS [23]. A significant majority of late-onset neonatal sepsis (86.8%) was observed in our study which corroborated with other studies from tertiary level neonatal units in South African where LOS accounted for 83.0 to 93.0% of cases [10–12].

Studies concluded that almost 70.0% of first-onset infections in LOS were caused by gram-positive organisms, followed by gram-negatives (18.0%) and fungi (12.0%) which is supported by the findings of this study [24]. Apart from CoNS, the leading organisms were enterococci, *K. pneumoniae* and *A. baumannii*. These findings are consistent with reports from other African countries and India [13, 14, 25]. Despite the evidence surrounding CoNS as pathogens of neonatal sepsis, isolates may still represent blood culture contamination as skin colonisers [26]. The absence of clinical data in this study rendered determining clinical significance challenging. Of note, studies from other low-and-middle income countries have also demonstrated that CoNS were leading pathogens of neonatal sepsis after adjusting for contamination [8, 10, 11, 13, 15, 25].

Studies from Botswana and South Africa have documented enterococci as a leading cause of gram-positive sepsis (12.2 to 18.0%) [15, 16]. In the current study, enterococci emerged as significant pathogens of gram-positive sepsis with a predominance of *E. faecium* in LOS and *E. faecalis* in EOS. The possibility remains that antibiotic selective pressure may drive the shift from ampicillin-susceptible *E. faecalis* in EOS to ampicillin-resistant *E. faecium* in LOS*.* This species-specific differentiation requires confirmation with larger studies.

South African studies determined *K. pneumoniae* and *S. aureus* are leading pathogens of neonatal sepsis [11, 12]. However, in this study, *S. aureus* was not a common cause of sepsis. Among Enterobacterales, *K. pneumoniae* was isolated most frequently, alongside other organisms such as *E. coli.* Studies confirm that *E. coli* and *K. pneumoniae* are well-recognized pathogens of neonatal sepsis [8, 9, 27]. Additionally, our study found that non-fermenters such as *A. baumannii*, *S. maltophilia* and *P. aeruginosa* were also causes of sepsis, which was supported by other reports [28, 29].

Though overall rates of candidaemia remained low when compared to bacteraemia, *C. parapsilosis* was a notable cause of neonatal candidaemia, an observation corroborated by findings from other South African studies [16, 30].

Currently, ampicillin, cloxacillin and vancomycin are advocated in the treatment regimen of gram-positive organisms. *Staphylococcus aureus* and CoNS showed high resistance to cloxacillin, which has been demonstrated both globally and in South Africa [10, 25, 31]. In addition, due to the prevalence of *E. faecium* within the NICU, ampicillin susceptibility was low, which was also described in another South African setting [10].

It has been observed that resistance amongst the gram-negative population is on the rise within NICUs [32]. This study confirms these findings, as gram-negative organisms in this unit demonstrated high levels of resistance to first-line antibiotics (i.e., cefotaxime and gentamicin). However, susceptibility to broader-spectrum agents, which included carbapenems and amikacin, was evident. Other research has reported high levels of resistance to antimicrobials used to treat gram-negatives, including ampicillin, ceftazidime, cefotaxime, imipenem, gentamicin, ciprofloxacin while maintaining susceptibility to meropenem, which was confirmed by our analysis [33]..

Neonatal units have demonstrated variable fluconazole susceptibility patterns that are dependent upon the species of their predominating fungal pathogens [34, 35]. The administration of fluconazole prophylaxis to high-risk patients, which is the practice within this NICU, may lead to selection of fluconazole resistant species [32]. Approximately half of all candida isolates, especially *C. parapsilosis,* were resistant to fluconazole and this finding is supported by another South African study that reported fluconazole resistance amongst *C. parapsilosis* of 54.0% [7].

When susceptibility trends over the three study periods were compared for all antibiotics, susceptibility of cefotaxime amongst Enterobacterales increased significantly during the study period, although it remained below 50.0%. This observation may be attributed to the decreasing levels of ESBL organisms. There were no statistically significant downward trends in susceptibility apart from amikacin. Literature includes mixed reports regarding the susceptibility of aminoglycosides, such as amikacin and gentamicin. Roy et al. (2017), reported increased amikacin resistance in an Indian NICU over a 15-year period [36]. In contrast, low levels of amikacin resistance have been documented in other studies; therefore, amikacin is frequently included in empiric regimens [37]. The temporal increase in susceptibility of gentamicin found in this study, may be attributed to the preferential use of amikacin, instead of gentamicin, in the unit. This occurrence requires further observation over time.

Despite the global trend towards increasing MDRO, the rates of ESBLs in our study have undergone a statistically significant decrease from 2014 to 2018, which is in keeping with the increased cefotaxime susceptibilities observed. Changes in ESBL rates may also be attributed to the increase of other MDROs within the unit. XDR *A. baumannii* were important isolates during the study period that showed an increased occurrence, albeit not statistically significant. This study also demonstrated the emergence of CRE, which has been implicated in neonatal sepsis in a recent South African study [33]. Although MRSA has been found to cause neonatal sepsis in other South African studies, the overall contribution of MRSA to the study population was lower than described in other South Africa neonatal settings [10, 12, 16].

According to the World Health Organization (WHO), first line antibiotic therapy for neonatal sepsis consists of benzylpenicillin/ampicillin and gentamicin [5]. Choosing an appropriate empiric antimicrobial regimen in the IALCH NICU remains a challenge. Amoxicillin-clavulanate and piperacillin-tazobactam as first-line agents for EOS and LOS, respectively, currently used in this NICU, are inappropriate. In view of the high levels of antimicrobial resistance observed, meropenem with or without vancomycin provides optimal empiric cover for both EOS and LOS. The addition of vancomycin would depend on the presence of risk factors for staphylococcal or *E. faecium* infection such as the presence of intra-abdominal sepsis. This regimen would be effective due to the high prevalence of ESBL organisms and resistant gram-positive organisms in the neonatal unit. However, it would not be active against XDR *A. baumannii.* Therefore, in clinically unstable cases with suspected preliminary *Acinetobacter* species, as suggested by the gram stain and MDR appearance on direct Kirby-Bauer disk diffusion, appropriate combination therapy with collaboration between clinician and clinical microbiologists is suggested. This approach would be on an individual basis and would require careful clinically correlation due to the inherent limitations of direct disk diffusion testing. Newer antimicrobials are available for the management of sepsis. However, further investigation into use in the neonatal population is required. For empiric treatment for candidiasis, amphotericin B is recommended, which correlates with recommendations issued by the Infectious Diseases Society of America [38].

Apart from using empiric broad-spectrum antimicrobials in the unit, effective infection control practices need to be enforced in curb the spread of MDROs. Consequently, several infection control measures are in effect within the unit such strict hand hygiene practices, isolation and cohorting of patients, intensive environmental cleaning, individual-use equipment, and the placement of a sentry at the entrance of the unit to monitor handwashing and wearing of personal protective equipment of all those who enter the unit. These measures have been in effect during the study period. In addition, antimicrobial stewardship practices are utilised, which include the de-escalation of therapy in conjunction with microbiological confirmation and the discontinuation of antimicrobials based on clinical, haematological, and microbiological grounds. Decisions regarding antimicrobials are made jointly with the microbiologist and the attending clinician.

When assessing the appropriateness of the above recommendations, the study’s limitations need to be considered. This study is based on laboratory surveillance and clinical data was not collected. However, clinical correlation is required to assist decisions regarding clinical relevance of potential pathogens in blood cultures, especially for CoNS. Results cannot be generalised to other settings, hospitals, or patient profiles as a single centre was utilised for the analysis. Premature neonates were not stratified within the study population and may present a different microbial profile than other groups of neonates. This study may be underpowered to determine temporal fluctuations amongst less frequently occurring organisms. Lastly, some antimicrobial data was unavailable.

## Conclusion

First-line antimicrobials, advocated by the WHO for treatment of neonatal sepsis, have proven ineffective in this unit due to high levels of AMR. Gram-positive sepsis, caused by CoNS and enterococci, was the leading cause of sepsis in this study. Gram-negative sepsis occurs to a lesser extent and is mainly comprised of XDR *A. baumannii* and ESBL *K. pneumoniae*. These MDROs create a therapeutic challenge and require broad-spectrum agents or combination therapy in consultation with paediatric infectious disease specialists and clinical microbiologists. The resistance noted in fungal isolates also necessitates broad-spectrum antifungals. Therefore, surveillance of the microbial profile of neonatal sepsis provides data to develop an appropriate empiric regimen and antimicrobial stewardship activities, which would ultimately improve sepsis outcomes.

## Data Availability

The data that support the findings of this study are available from the National Health Laboratory Service (Sandringham, South Africa) but restrictions apply to the availability of these data, which were used under license for the current study, and so are not publicly available. Data are however available from the authors upon reasonable request and with permission of the National Health Laboratory Service (Sandringham, South Africa).

## Abbreviations

AMR: Antimicrobial resistance
CoNS: Coagulase-negative staphylococci
CRE: Carbapenem-resistant Enterobacterales
EOS: Early-onset sepsis
ESBL: Extended-spectrum beta-lactamase
GBS: Group B streptococcus
HIC: High-income country
IALCH: Inkosi Albert Luthuli Central Hospital
LOS: Late onset sepsis
MDR: Multi-drug resistant
MDRO: Multidrug resistant organism
MRSA: Methicillin-resistant *Staphylococcus aureus*
NICU: Neonatal intensive care unit
OR: Odds ratio
VRE: Vancomycin resistant enterococci
WHO: World Health Organization
XDR: Extensively-drug resistant
